# Influence of the Mass Media and Body Dissatisfaction on the Risk in Adolescents of Developing Eating Disorders

**DOI:** 10.3390/ijerph16091508

**Published:** 2019-04-29

**Authors:** Francisco Nataniel Macedo Uchôa, Natalia Macêdo Uchôa, Thiago Medeiros da Costa Daniele, Romário Pinheiro Lustosa, Nuno Domingos Garrido, Naira Figueiredo Deana, Ágata Cristina Marques Aranha, Nilton Alves

**Affiliations:** 1 Department of Sports Sciences, Exercise and Health, Trás-os-Montes and Alto Douro University, 5001-801 Vila Real, Portugal; nataniel4@hotmail.com (F.N.M.U.); ngarrido@utad.pt (N.D.G.); aaranha@utad.pt (Á.C.M.A.); 2 Department of Physiotherapy, University Center of Grande Fortaleza, Unigrande, Fortaleza 60525-571, Brazil; nataliamacedouchoa@hotmail.com; 3 Department of Clinical Medicine, Federal University of Ceara, Fortaleza, Ceará 60430-275, Brazil; danielethiago@yahoo.com.br; 4 Higer Institute of Biomedical Sciences, State University of Ceará, Fortalea, Ceará 60741-000, Brazil; romario-lustosa@hotmail.com; 5 Research Center in Sports Sciences, Health Sciences & Human Development (CIDESD), 4780000 Vila Real, Portugal; 6 Department of Sports Sciences, University of Beira Interior, 6201-001 Covilhã, Portugal; 7 Center for Research in Epidemiology, Economics and Oral Public Health (CIEESPO), Faculty of Dentistry, Universidad de La Frontera, Temuco 4780000, Chile; n.figueiredo01@ufromail.cl; 8 Applied Morphology Research Center (CIMA), Faculty of Dentistry, Universidad de La Frontera, Temuco 4780000, Chile; 9 Center of Excellence in Surgical and Morphological Research (CEMyQ), Faculty of Medicine, Universidad de La Frontera, Temuco 4780000, Chile

**Keywords:** body image, mass media, eating disorders, adolescents

## Abstract

Media influence may lead adolescents to internalize patterns of physical beauty, resulting in dissatisfaction with their own bodies when they are unable to match up to these patterns. In the constant search for an ‘ideal body’, adolescents may begin to develop risk behaviors for the development of eating disorders (ED). The object of this study was to analyze the influence of the mass media on body dissatisfaction (BD) and on ED in adolescents, comparing genders. We also analyzed the influence of BD on the risk of developing unsuitable eating behaviors, with risk of ED, comparing genders. A cross-sectional study was carried out with 1011 adolescents: 527 girls and 484 boys. The BMI of each adolescent was determined, and the instruments EAT-26, Sociocultural Attitudes towards Appearance Questionnaire-3 (SATAQ-3), and body shape questionnaire (BSQ), were applied. For statistical analysis, we used Student’s *t*-test, the chi-square test, Pearson’s correlation test, the odds ratio, and hierarchical multiple linear regression. The influence of the mass media is associated with a greater probability of adolescents presenting BD. An increase in BD is associated with an increased risk of developing ED in adolescents of both genders but is greater in girls than in boys. Furthermore, the influence of the MM and BMI are predictors of BD in both genders; and BD is a predictor of ED risk in both girls and boys.

## 1. Introduction

Body image (BI) refers to the perceptions, thoughts, and feelings of individuals with respect to their bodies [1]. Internalization of ideals of beauty leads individuals to estimate the difference between their own bodies and this ideal, triggering BD when this ideal cannot be achieved [2]. BD among adolescents is a serious public health problem because it generates a series of damaging conditions [3,4], such as compromised emotional well-being, low self-esteem, symptoms of depression, and disordered eating [3]. BD affects adolescents in different ways: Boys tend to suffer sociocultural pressure encouraging them to have bigger, more muscular bodies [5], while girls are under pressure to have a thin or ultra-thin body [6]. The pressure may come from parents, friends, dating partners or even from the media, which appear to exercise a strong influence on adolescents [7]. Some sociocultural factors may contribute to the development of risk behaviors among adolescents [8]. The social pressure generated by the communication mass media (MM) propagates the idea that it is necessary to achieve an ‘ideal of beauty’, emphasizing thinness for women and muscular bodies for men [9]; the MM also often associate professional and personal success with the ‘perfect body’ [10]. The media disseminate a pattern of beauty which may be difficult to achieve for many people [11,12]; internalization of this ideal of beauty appears to play a mediating role between sociocultural pressure and the development of BD [13]. A distorted image of the ‘ideal body’ associated with a feeling of dissatisfaction with oneself may lead to disordered eating habits, compromising adolescents’ health [14].

Grabe et al. [15], in a meta-analysis, observed that women and adolescents exposed to the media spent more money on improving their appearance and were more susceptible to eating disorders (ED) because they felt dissatisfied with their body image. High susceptibility to developing unsuitable eating behaviors may be related with an attempt to achieve a pattern of beauty promoted by the MM. Skipping meals, exercising obsessively, causing vomiting, and using laxatives or diuretics have been recognized as subclinical symptoms of ED [16]. Adolescence is a period of vulnerability, especially for girls. This can determine a greater risk of developing ED as adolescents begin to focus on their body image during this stage as a result of changes related with puberty, including a sudden weight increase [17]. In industrialized countries, ED are the third most common chronic disease in female adolescents [18]. In Western countries, the rate of anorexia (AN) is 0.3% and of bulimia (BN) 1% [19]. Previous studies have shown that BD is one of the most consistent risks for developing eating disorders (ED) [20,21] and is one of the 10 principal causes of disability in young women [22]. BD and ED may be determined by different variables and are strongly related with social and cultural norms and ethnic group [23]; the causes of ED in each population therefore need to be identified. Eating behavior disorders (anorexia nervosa and bulimia nervosa) are mental health problems which affect individuals of both genders, but principally young women [24].

One way of identifying the risk factors which may lead to BD or the risk of developing ED, or identifying the internalization of sociocultural factors, is by applying self-reporting instruments. Many instruments have been created to measure sociocultural factors, one of the most-used being the Sociocultural Attitudes towards Appearance Questionnaire-3 (SATAQ-3), which measures the influence of the communication media on body image [25,26]. BD can also be measured by instruments; the body shape questionnaire (BSQ) is a self-reporting instrument which assesses the individual’s concern with body shape and weight [27]. Easily applied self-reporting instruments are useful for identifying individuals at risk of ED [28]; the individual can then be referred to a specialist for early diagnosis and appropriate treatment. The Eating Attitudes Test-26 (EAT-26) is a very reliable and sensitive multidimensional instrument for early detection of individuals with ED [28]. Identifying the factors which cause adolescents to feel dissatisfied with their body image and the factors which increase their susceptibility to the development of ED is important for the creation of prevention and support programs for these adolescents. The objectives of this study were therefore: (1) To analyze the influence of the mass media on BD and ED, comparing boys and girls; and (2) to analyze the influence of BD on ED, comparing boys and girls.

## 2. Method

### 2.1. Participants

We carried out a descriptive, observational, cross-sectional study. The sample size was calculated from the population of the city of Fortaleza, Brazil, with 93,837 secondary school students in public and private schools [29]. We used the formula $n_{0}=\frac{1}{E_{0}^{2}}$, where $n_{0}$ is the first approach to the sample size and $E_{0}^{2}$ is the tolerable sample error. With a sample error of 4%, *n* was 621 students; our total sample was larger. A total of 1300 adolescents of both genders, aged between 14 and 18 years, from 16 secondary schools in the city of Fortaleza, Brazil, were invited to participate in the study; 1011 adolescents (527 girls and 484 boys) completed and handed in the instruments. The recruitment of subjects and the application of the instruments were carried out in August–November 2017.

### 2.2. Demographic and Anthropometric Measures

The equipment used to take anthropometric measurements was an electronic scale (*Filizola*^®^-110), a stadiometer (*Sanny*^®^), and an anthropometric tape (*Sanny*^®^). To assess body fat, the body mass index (BMI) was used, defined as the body mass divided by the square of the body height, expressed in units of kg/m^2^ and analyzed by gender. BMI was calculated by the following formula: BMI = mass/height^2^. The BMI diagnosis was subdivided into accentuated thinness (BMI 16.0–16.99), thinness (BMI 17.00–18.49) normal (BMI 18.5–24.9), overweight (BMI 25.0 and 29.9), and obese (BMI ≥ 30.0).

### 2.3. Measurements

The Sociocultural Attitudes towards Appearance Questionnaire-3 (SATAQ-3) was developed to refine the measurement of internalization as well as to evaluate the distinctiveness and utility of other dimensions of media influence [26]. This questionnaire has been used previously in a Brazilian population, and its validity has been examined [30]. It consists of 30 items to measure four dimensions of media influence: Subscale 1—Internalization–General (INT-GEN); Subscale 2—Internalization–Athletic (INT-ATH); Subscale 3—Pressure (PRESS); and Subscale 4—Information (INFO). The two internalization subscales evaluate the incorporation of appearance norms promoted by the media into individuals’ own identities, up to the point at which they desire or strive to match the ideals. INT-GEN was classified as low (≤23 points), middle (23–28 points), and high (≥29 points). INT-ATH was classified as low (≤12 points), middle (13–16 points), and high (≥17 points). The PRESS subscale contains items that index a subjective sense of pressure from exposure to media images and messages to modify one’s appearance. PRESS was classified as low (≤15 points), middle (16–21 points), and high (≥22 points). The INFO subscale captures the recognition that information regarding appearance norms is available from media sources [31]. INFO was classified as low (≤26 points), middle (27–30 points), and high (≥31 points). The result is given by the sum of the responses from each element; the higher the score, the greater the internalization of the media messages specific to the element. The total score for mass media influence was classified as: Small influence (≤77 points), moderate influence (78–94 points), and large influence (≥95 points).

The body shape questionnaire (BSQ) is an instrument designed to measure dissatisfaction with body image [32]. A Brazilian version of the BSQ for adolescents of both genders has been previously validated [27].

The questionnaire contains 34 questions on a 6-point Likert scale, ranging from 1 (never) to 6 (always), spread over 4 subscales: 1. Self-perception of body shape (22 questions); 2. comparative concern (5 questions); 3. attitude (5 questions), and 4. severe alterations (2 questions), to give a score ranging between 34 and 204 points. The higher the score, the greater the dissatisfaction. Based on their BSQ results, the subjects were divided into four levels of dissatisfaction with physical appearance, following the model validated for Brazilian adolescents by Conti and Latorre [27]: Score ≤79 (no dissatisfaction with BI); 80 ≤ score ≤ 109 (slight dissatisfaction with BI); 110≤ score ≤ 140 (moderate dissatisfaction with BI); score ≥ 140 (severe dissatisfaction with BI).

The Eating Attitudes Test (EAT-26) is a self-reporting questionnaire which analyzes unsuitable eating behaviors; it has been validated for Brazilians [33,34]. It contains 26 items with answers on a Likert-type scale (0 = never, hardly ever or occasionally; 1 = sometimes; 2 = often; 3 = always). There are three subscales to evaluate the impact of environmental and social factors on food ingestion: 1. Diet, related with pathological rejection of foods with high calorie content and concern with physical appearance; 2. bulimia and concern with foods, referring to episodes of compulsive eating followed by purgative behavior for bodyweight loss/control; 3. oral self-control, referring to self-control with respect to food. Scores ≥ 20 indicate risk behavior for triggering eating disorders.

### 2.4. Procedure

Initially, the schools were contacted; once authorization had been obtained, recruitment of adolescents started. Informative sessions were held to explain to the adolescents the nature of the study and what their participation would be. Once the adolescents had agreed to participate, an informed consent was signed by both them and their parents/guardians. Scales to assess the influence of the media (SATAQ-3), level of satisfaction with body image (BSQ), and eating attitudes (EAT-26) were then applied to the participants. The instruments were completed individually during school time, and anonymity was maintained. The adolescents remained in their classrooms with one of the research team present to answer any doubts about how to complete the questionnaires.

In addition, the BMI of each adolescent was calculated. The result of each instrument was analyzed. The correlations between media influence × body image; media influence × eating attitudes; media influence × BMI, body image × eating attitudes risk were calculated. For this study, a comparison was made of the results between genders.

### 2.5. Data Analysis

Descriptive statistics are presented as mean ± standard deviation (±SD), range, and frequency (% values). Student’s *t*-test was used for comparison between the genders. A chi-square test was used for qualitative variables. Pearson’s correlation and the odds ratio were applied between the variables analyzed. Hierarchical multiple linear regression was applied to find whether MM influence (SATAQ-3), BD (BSQ), and BMI are able to predict the development of ED (EAT-26). Hierarchical multiple linear regression was also used to investigate whether BMI and MM (SATAQ-3) can predict BD (BSQ). Statistical analysis was carried out using SPSS for Windows, version 20.0 (IBM Corp: Armonk, NY, USA). Statistical significance was set at *p* ≤ 0.05.

### 2.6. Ethical Approval

All procedures performed in studies involving human participants were in accordance with the ethical standards of the institutional and/or national research committee and with the 1975 Helsinki declaration and its later amendments or comparable ethical standards. Informed consent was obtained from all individual participants included in this study. Adolescents were excluded if their parents refused their informed consent. This study was carried out in accordance with the Helsinki Declaration and was approved by the Ethics Committee for Research on Human Beings, file number 2.193.376.

### 2.7. Data Availability

The data and materials supporting the conclusions of this manuscript are included in the article.

## 3. Results

Table 1 shows the general characteristics of the adolescents separated by gender. A total of 54.7% of the adolescents suffered a slight influence from the MM, 27.3% a moderate influence, and 18.0% a strong influence. A larger number of girls than boys was found to suffer a strong influence from the MM (Table 2). We observed that the values for all the subscales of SATAQ-3 were higher for girls than boys, showing that they are more strongly influenced by the media. Both genders presented moderate influence in INT-GEN and low influence in INT-ATH and INFO. In the PRESS subscale, the girls presented significantly higher values than the boys, experiencing moderate influence (Table 1).

A total of 28.7% of the adolescents presented BD: 15.1% slight BD, 7.8% moderate BD, and 5.7% severe BD (Table 2). A higher percentage of girls (19.6%) presented some degree of BD than boys (9%); boys presented a lower score than girls in the BSQ, showing greater body satisfaction (Table 1). A total of 73.7% of the adolescents presented normal BMI. Overweight was slightly more frequent in girls while obesity was slightly more frequent in boys (Table 2). A total of 6.8% of the adolescents presented unsuitable eating behaviors with risk of developing ED; the percentage was significantly higher in girls than in boys (Table 2). Furthermore, girls presented higher scores than boys in all the subscales of EAT-26 (Table 1).

### 3.1. Media Influence × BMI

Greater media influence was observed in boys with overweight and obesity than normal-weight and thin boys. A total of 85% of thin girls presented low media influence; greater influence was observed when their weight was higher, with 56% of overweight girls and 63% of obese girls feeling that they suffered moderate or high media influence. A very low significant positive correlation was observed between BMI and SATAQ-3 (Table 3).

### 3.2. Media Influence × Eating Attitudes

The percentage of boys and girls with score <20 in EAT-26 who felt that they were not influenced by the MM was over 60%. It was observed that adolescents with score >20 in EAT-26 felt influenced by the MM (Table 3). We also observed that boys who are influenced by the mass media have a four times higher likelihood of presenting risk behaviors for developing ED, while girls so affected had a three times higher likelihood of presenting risk behaviors for developing ED (Table 4).

### 3.3. Media Influence × Body Image

We observed that BD increased with increasing media influence, and there was a significant moderate positive concordance for adolescents of both genders (Table 3). Media influence determined that boys had a seven times higher likelihood of presenting body dissatisfaction, while in girls the figure was eight times higher (Table 4).

### 3.4. Body Image × Eating Attitudes

We observed that the majority of the adolescents, of both genders, who presented some degree of BD (slight, moderate or severe) presented unsuitable eating behaviors (Table 3). Pearson’s correlation showed that the presence of ED increases when BD is greater, with significant moderate positive concordance for boys and high for girls (Table 5). Body dissatisfaction determines a higher likelihood of developing ED: 16.04 times higher in girls and 12.89 times higher in boys (Table 4).

### 3.5. Body Image × BMI

BD was greater in girls who presented overweight and obesity than in normal-weight or thin girls (Table 3). A significant moderate positive correlation was observed between increased weight and BD for both genders (Table 5). Overweight and obese adolescents were 3 and 4 times more likely to present BD than thin or normal-weight adolescents (Table 4).

### 3.6. Predictors of Body Dissatisfaction

For girls, the regression model was statistically significant (F(2.524) = 180,859; *p* = 0.000; R^2^ = 0.406). The MM measured by SATAQ-3 (ß = 0.448; *t* = 13.146; *p* = 0.000) and the BMI (ß = 0.386; *t* = 11.311; *p* = 0.000) predicted BD in girls.

For boys, the regression model was statistically significant (F(2.481) = 121,232; *p* = 0.000; R^2^ = 0.335). The MM measured by SATAQ-3 (ß = 0.411; *t*=11.034; *p* = 0.000) and the BMI (ß = 0.390; *t* = 10.465; *p* = 0.000) predicted BD in boys.

### 3.7. Predictors of Eating Disorders

For girls, the regression model was statistically significant (F(1.525) = 404,710; *p* = 0.000; R^2^ = 0.435). The BD measured by the BSQ (ß = 0.660; *t* = 20.117; *p* = 0.000) was able to predict the development of ED in girls. The variables BMI and MM were not predictors and were excluded from the model.

For boys, the regression model was statistically significant (F(2.481) = 135,204; *p* = 0.000; R^2^ = 0.360). The BD measured by the BSQ (ß = 0.539; *t* = 13.355; *p* = 0.000) and media influence measured by SATAQ-3 (ß = 0.119; *t* = 2.944; *p* = 0.003) predicted the development of ED in boys. BMI was not a predictor and was excluded from the model.

## 4. Discussion

Media influence can lead adolescents to internalize the ideals imposed by society as desirable for themselves, increasing the probability that they will suffer BD and present disordered eating behaviors [35,36]. In the present investigation, 45.3% of the adolescents were moderately or strongly influenced by the media, with more girls (25.7%) influenced than boys (19.6%). Our findings coincided with those reported by van den Berg et al. [37], who indicate that girls presented internalization of an ideal body and perceived a greater pressure from the media than boys. The body image of the girls is significantly more negative when they are subjected to viewing ‘thin’ images than when they view ‘plus size’ models or other types of image [38], suggesting that the thin figures of the models who are patterns of beauty contribute to a negative self-image [39]. Boys appear less susceptible to perceiving the pressure of the presentation of idealized images of muscular men, contributing to a lower level of BD than is found in girls [37]. Concern over body image may start at the beginning of adolescence, increase during that period, and diminish in young adults [40], affecting psychological well-being in different phases of life [41]. The kind and degree of body image disorder varies with age, ethnic group, peers, family, and sociocultural influences [42]. Sociocultural influences have been considered the strongest determining factors for developing a negative body image disorder [43] and a predictor of ED due to acceptance of the ‘thin ideal’ by adolescents [44]. Previous studies in Brazilian populations reported that 19.5% [45] and 30% [46] of adolescents presented BD; in the present study, 28.6% of the adolescents presented dissatisfaction with their bodies. It has been reported that the media play a bigger role for girls than boys in sociocultural messages about the body [37,47,48]; this was corroborated in the present study, in which BD was significantly lower in boys (9%) than girls (19.6%). A study in Egyptian female university students found more significant values, with 77.9% of the participants dissatisfied with their images [49]; the higher values found in Egyptian women may be due to the fact that a higher proportion were overweight or obese than in the present study. It should be noted that in the present research, high levels of media influence (measured by SATAQ-3) were related with higher levels of BD (measured by BSQ) in adolescents, corroborating previous studies [49] and showing that the negative influence of the media can affect adolescents’ self-image. Girls and boys influenced by the mass media are 7 to 8 times more likely to present BD, showing that self-image can be affected in both genders by a negative influence of the mass media. In addition, the hierarchical multiple linear regression analysis showed that MM influence and BMI are predictors of BD, corroborating previous studies [7,37,48,49,50]. In an earlier study carried out in girls, Mostafa et al. [49] showed that BMI was the strongest predictor of BD; however, in the present study, MM was a stronger predictor than BMI. In another study, Jaeger and Câmara [50] showed that BMI and the influence of MM were good predictors of BD with higher variance values than were found in the present study. Mellor et al. [47] said that BMI was a good predictor of BD in Malaysian boys, as well as the messages promoted by adults to lose weight and media messages to increase muscle size; in Malaysian girls, the messages of adults and the media to lose weight, and the messages of classmates to put on muscle and reduce weight were predictors of BD [47]. Although girls generally present greater BD than boys [37], it should be noted that MM and BMI were variables which predicted BD in both genders, showing that boys also internalize media messages—albeit to a lesser extent—reflected in greater dissatisfaction with their body image. Jaeguer and Câmara [50] stressed the role of MM in BD, showing that the presentation in the media of specific body types may have a drastic effect on the quality of life of individuals, resulting in increased BD.

Current studies also suggest that exposure to the media is related with an increased tendency to start unhealthy eating behaviors [51]. ED, such as AN and BN, are problems which primarily affect young women; they become severe, almost always chronic disorders, with high levels of mortality and dysfunctionality [52]. Approximately 0.5% of adolescents are diagnosed with AN and 3% with BN, of whom 90 and 95% respectively are women [53]. In the present investigation, we found that the increase in BD was associated with an increased risk of developing ED, the risk being higher in girls than in boys, corroborating previous studies [54]. It should be noted that the present study was carried out away from the clinical environment, in adolescents who had not been diagnosed with ED; we found that 6.8% of our sample presented risk behaviors for developing ED. Similar percentages to those found in the present study were reported for Koreans, with 6.2% of adolescents presenting risk behaviors for developing ED [40]; higher percentages were reported for Peruvians, at 13.2% [55], Spanish adolescents, at 10.2% [51], and another study in Brazilians at 17.4% [34]. The risk of developing ED was significantly higher in girls than boys, corroborating previous findings [51]. By contrast, Jung et al. [40] reported that there were no differences between genders in Korean adolescents. Lazo-Montoya et al. [55] stated that the risk of unsuitable eating habits, and thus of developing ED, increases when the influence of the MM is greater, corroborating the findings of the present study. In an earlier study, Furnham et al. [9] observed that males and females presented comparable degrees of BD, but in opposite directions: 73% of males wanted to be heavier and 41% thinner; while 22% of females wanted to increase their weight and 63% wanted to reduce their physical size [9]. Thus, while underweight males appear to be dissatisfied with their bodies, underweight females appear to be very satisfied [56]. Both genders desire physical perfection, but it appears to be less difficult to increase weight by vigorous exercise and weight-training, and not just by diet; whereas achieving the patterns of female beauty imposed by the media, of extreme thinness, may cause girls to start unsuitable eating behaviors, increasing their risk of developing ED [57]. Many individuals who suffer eating behavior disorders are not diagnosed [28], so they do not receive the necessary support and training for their condition. We note that EAT-26 is not a diagnostic tool, but it is a reliable and sensitive tool for identifying ED [28]; this is important for early detection of ED and referral of at risk adolescents to specialists who can monitor and treat them.

In the present study, BD was the principal predictor for the development of ED in boys and girls, corroborating the findings of previous studies [58,59]. Fortes et al. [59] showed that BD accounts for 51% of the variance of ED. In another study, Fortes et al. [58] showed that BD accounts for 59% of the variance in girls and 47% in boys; these values are rather higher than those found in our study, of 43.4% for girls and 34.7% for boys. The higher percentage found for girls may be due to the fact that they have internalized the ‘thin ideal’ more strongly than boys [60]. Another prediction factor for the development of ED was media influence; however, this finding was reported only for boys and showed less predictive power than BD. Tiggemann et al. [61] reported that they found no relation between MM influence and disordered eating behaviors. Lai et al. [36] noted that sociocultural attitudes are a significant predictor for disordered eating; they state that there is a need for professionals to re-examine understanding of the impact of sociocultural attitudes on BD and eating disorders. Argyrides and Kkeli [62] state that the internalization of the ‘thin ideal’ was a predictor for the development of ED in female university students; however, it should be noted that this variable in isolation presented low predictive power. The different findings reported in the literature may be related with the fact that the studies were carried out in different populations, in which individuals may have internalized sociocultural influences and MM pressure differently. Furnhan et al. [9] and Tiggemann et al. [61] say that body mass can also predict ED, but this finding was not corroborated by the present study, in which we observed that BMI cannot predict ED. Argyrides and Kkeli [62], in a study in female university students, reported a similar result to ours, showing that BMI is not a predictor of ED. However, it should be noted that although BMI is not a predictor of ED, it is a strong predictor of BD which in turn is related with—and may be a predictor of—ED. Thus, BMI is a variable which needs to be taken into consideration when analyzing adolescents at risk of developing ED.

Adolescence is a period in which individuals are psychologically very vulnerable, and it is important to give them the necessary support to enable them to develop into healthy adults. Adolescents need to be taught to filter the information they receive from the mass media in order to prevent the negative impact that the media may have. Interventions in the literature dealing with the mass media aim to improve individuals’ ability to access, analyze, evaluate, and create media [63]; they are recommended for developing critical thought with the object of reducing vulnerability to negative influence by the media [64]. In an earlier study, Posavac et al. [65] observed that female students with a negative self-image who were given psychoeducational instruction involving media analysis were less likely to make social comparisons and were less susceptible to the negative influence of images of ‘thin’ beauty than students who received no such instruction. Individuals with a positive body image tend to filter and reject unreal images in the media to protect their own body image [66].

The present study shows that MM influence is associated with a higher probability of presenting BD and developing ED. Media influence increases the chances of BD, and BD increases the risk of ED. Furthermore, we note that MM, BD, BMI, and unsuitable eating behaviors are interrelated variables; MM and BMI are predictors of BD, and BD in turn is an important predictor of risk of ED in both girls and boys. Identifying the risk factors for ED is important for orienting the creation of prevention programs which will support the specific needs of adolescents, giving them the ability to think critically about the information they receive in order to diminish the impact caused by the negative influence of the mass media.

### Study Limitations

One limitation of our study is that SATAQ-3 measures the influence of the media, including television, cinema, and magazines; however, it does not measure the influence of the internet and social networks which are now so strongly present in the lives of adolescents. SATAQ-4 could not be used because at the time of data collection for the present study, the Portuguese version of this instrument had not been validated. We must add that we did not collect information on the frequency with which the adolescents watched television or read magazines, or the time they devoted to these activities. The internalization of the messages disseminated by the media may be greater in those who spend more time in contact with the communication media; however, this aspect could not be analyzed in the present study. We also note that the adolescents who took part in this study were not evaluated by a specialist doctor to confirm the presence or absence of ED. Another limitation is that the study design measured only the answers at the moment of enquiry, and no temporal associations could be made. It should be noted that the sample included in this study was representative of the city of Fortaleza; however, it is not representative for Brazil, and studies should be carried out in other large population centers as the type of pressure affecting adolescents may differ.

## 5. Conclusions

The influence of the mass media is associated with a greater probability of adolescents presenting BD. An increase in BD is associated with increased risk of developing ED in adolescents of both genders, but the risk is greater in girls than in boys. Media influence increased the chances of the presence of BD, and BD increased the risk of ED in both boys and girls. Furthermore, MM influence and BMI are predictors of BD; and BD is a predictor for risk of ED in both boys and girls. We recommend the creation of prevention programs to minimize the negative impact of the mass media, improving self-image and reducing the risk of ED among adolescents.

## Figures and Tables

**Table 1:** Comparison of quantitative variables in adolescents aged between 14 and 18 years, by gender.

Variables		Values	Gender	
			Girls	Boys
Age (years)		Mean (SD)	15.6 (1.0)	15.7 (1.1)
		*p* value	0.105	
BMI		Mean (SD)	21.8 (3.5)	22.0 (3.7)
		*p* value	0.468	
Body image (score) (BSQ)		Mean (SD)	78.6 (34.9)	63.0 (27.3)
		*p* value	≤0.001	
Eating attitudes (score) (EAT-26)	Diet	Mean (SD)	7.74 (7.61)	5.12 (5.58)
		*p* value	≤0.001	
	Bulimia	Mean (SD)	3.18 (2.97)	2.08 (2.54)
		*p* value	≤0.001	
	Oral control	Mean (SD)	4.41 (3.86)	3.57 (3.36)
		*p* value	≤0.001	
	Total	Mean (SD)	15.3 (10.9)	10.7 (8.6)
		*p* value	≤0.001	
Media influence (SATAQ-3)	INT-GEN	Mean (SD)	24.4 (8.4)	23.2 (7.4)
		*p* value	0.011	
	INT-ATH	Mean (SD)	12.0 (4.7)	12.7 (4.9)
		*p* value	0.016	
	PRESS	Mean (SD)	17.4 (7.2)	14.9 (6.0)
		*p* value	≤0.001	
	INFO	Mean (SD)	25.1 (7.1)	24.3 (6.5)
		*p* value	0.088	
	Total	Mean (SD)	81.2 (23.3)	77.2 (20.5)
		*p* value	0.004	

SD: Standard deviation.

**Table 2:** Classification of adolescents for eating disorders risk, body mass index (BMI), body dissatisfaction, and mass media influence, by gender.

Instruments	Classification/*p* Value	Girls	Boys	Total	*p* Value
Eating attitudes (EAT-26)	No ED risk	46.9%	46.3%	93.2%	≤0.001
	ED risk	5.4%	1.5%	6.8%	
BMI	Accentuated thinness	0.3%	1.0%	1.3%	0.006
	Thinness	1.0%	0.4%	1.4%	
	Normality	39.3%	34.4%	73.7%	
	Overweight	9.3%	8.0%	17.3%	
	Obesity	1.1%	2.8%	3.9%	
	Severe obesity	1.2%	1.3%	2.5%	
Body image (BSQ)	No dissatisfaction	32.4%	38.9%	71.3%	≤0.001
	Slight dissatisfaction	9.9%	5.2%	15.1%	
	Moderate dissatisfaction	5.2%	2.6%	7.8%	
	Severe Dissatisfaction	4.5%	1.2%	5.7%	
Media influence (SATAQ-3)	Low	26.4%	28.3%	54.7%	≤0.001
	Moderate	14.0%	13.3%	27.3%	
	High	11.7%	6.3%	18%	

**Table 3:** Classification of adolescents by EAT-26, body shape questionnaire (BSQ), and BMI and relation with SATAQ-3.

Instruments	Score/*p* Value	SATAQ-3					
		Boys (*n* = 484)			Girls (*n* = 521)		
		Low	Moderate	High	Low	Moderate	High
Eating attitudes (EAT-26)	ED risk	32%	37%	31%	29%	32%	39%
	No ED risk	67%	23%	11%	60%	23%	17%
	*p* value	<0.001			<0.001		
	Pearson’s correlation	0.350 (*p* ≤ 0.001)			0.387 (*p* ≤ 0.001)		
Body image (BSQ)	No dissatisfaction	66.5%	25.8%	7.7%	65.7%	21.0%	13.4%
	Slight dissatisfaction	32.1%	39.6%	28.3%	35.0%	43.0%	22.0%
	Moderate dissatisfaction	23.1%	34.6%	42.3%	18.9%	35.8%	45.3%
	Severe Dissatisfaction	18.2%	27.3%	54.5%	15.2%	23.9%	60.9%
	*p* value	≤0.001			≤0.001		
	Pearson’s correlation	0.429 (*p* ≤ 0.001)			0.515 (*p* ≤ 0.001)		
BMI	Thinness	57%	36%	7%	85%	8%	8%
	Normality	64%	24%	11%	55%	26%	20%
	Overweight	60%	23%	16%	40%	27%	33%
	Obesity	51%	24%	24%	39%	26%	35%
	*p* value	0.288			0.010		
	Pearson’s correlation	0.046 (*p* ≤ 0.001)			0.158 (*p* ≤ 0.001)		

ED: Eating disorders.

**Table 4:** Odds ratio (OR) for the relation between SATAQ-3 and other variables, and EAT-26 and other variables.

Instruments	Girls		Boys	
	OR	CI 95%	OR	CI 95%
Media influence × Eating attitudes	3.60	2.36–5.50	4.22	2.42–7.38
Media influence × BMI	1.50	1.0–2.23	1.33	0.89–1.99
Media influence × Body image	7.32	4.19–12.78	6.11	2.82–13.23
Body image × Eating attitudes	16.04	9.55–26.95	12.89	6.29–26.39
Body image × BMI	3.01	1.68–5.39	4.39	2.54–7.60

CI: Confidence interval.

**Table 5:** Classification of adolescents by BMI and EAT-26 subscales, and their relation with BSQ.

Instruments	Classification	BSQ							
		Boys				Girls			
		ND	SD	MD	SeD	ND	SD	MD	SeD
Eating attitudes (EAT-26)	ED risk	38%	28%	22%	12%	17%	29%	22%	32%
	No ED risk	88%	8%	3%	1%	78%	15%	6%	1%
	*p* value	≤0.001				≤0.001			
	Pearson’s correlation	0.590 (*p* ≤ 0.001)				0.659 (*p* ≤ 0.001)			
BMI	Thinness	86%	14%	0%	0%	85%	8%	8%	0%
	Normality	88%	8%	3%	1%	69%	18%	8%	5%
	Overweight	64%	20%	11%	5%	36%	27%	15%	22%
	Obesity	59%	20%	15%	7%	39%	9%	22%	30%
	*p* value	0.288				0.010			
	Pearson’s correlation	0.408 (*p* ≤ 0.001)				0.452 (*p* ≤ 0.001)			

ND = no dissatisfaction, SD = slight dissatisfaction, MD = moderate dissatisfaction, SeD = severe dissatisfaction.
